# The impact of maternal gestational weight gain on cardiometabolic risk factors in children

**DOI:** 10.1007/s00125-018-4724-x

**Published:** 2018-09-17

**Authors:** Claudia H. T. Tam, Ronald C. W. Ma, Lai Yuk Yuen, Risa Ozaki, Albert Martin Li, Yong Hou, Michael H. M. Chan, Chung Shun Ho, Xilin Yang, Juliana C. N. Chan, Wing Hung Tam

**Affiliations:** 1 9/F, Lui Che Woo Clinical Science Building, Department of Medicine and Therapeutics, The Chinese University of Hong Kong, Prince of Wales Hospital, 30–32 Ngan Shing Street, Shatin, Hong Kong Special Administrative Region Hong Kong; 2 Hong Kong Institute of Diabetes and Obesity, The Chinese University of Hong Kong, Shatin, Hong Kong Special Administrative Region Hong Kong; 3 Li Ka Shing Institute of Health Sciences, The Chinese University of Hong Kong, Shatin, Hong Kong Special Administrative Region Hong Kong; 4 1/F, Special Block (Block E), Department of Obstetrics and Gynaecology, The Chinese University of Hong Kong, Prince of Wales Hospital, 30–32 Ngan Shing Street, Shatin, Hong Kong Special Administrative Region Hong Kong; 5 Department of Paediatrics, The Chinese University of Hong Kong, Shatin, Hong Kong Special Administrative Region Hong Kong; 6 Department of Chemical Pathology, The Chinese University of Hong Kong, Shatin, Hong Kong Special Administrative Region Hong Kong; 7 Department of Epidemiology and Biostatistics, School of Public Health, Tianjin Medical University, Tianjin, China

**Keywords:** Adiposity, Cardiometabolic risk factors, Chinese children, Gestational weight gain, Hypertension, Insulin resistance, Maternal pre-pregnancy BMI

## Abstract

**Aims/hypothesis:**

Accumulating evidence suggests an impact of gestational weight gain (GWG) on pregnancy outcomes; however, data on cardiometabolic risk factors later in life have not been comprehensively studied. This study aimed to evaluate the relationship between GWG and cardiometabolic risk in offspring aged 7 years.

**Methods:**

We included a total of 905 mother–child pairs who enrolled in the follow-up visit of the multicentre Hyperglycemia and Adverse Pregnancy Outcome study, at the Hong Kong Centre. Women were classified as having gained weight below, within or exceeding the 2009 Institute of Medicine (IOM) guidelines. A standardised GWG according to pre-pregnancy BMI categories was calculated to explore for any quadratic relationship.

**Results:**

Independent of pre-pregnancy BMI, gestational hyperglycaemia and other confounders, women who gained more weight than the IOM recommendations had offspring with a larger body size and increased odds of adiposity, hypertension and insulin resistance (range of *p* values of all the traits: 4.6 × 10^−9^ < *p* < 0.0390) than women who were within the recommended range of weight gain during pregnancy. Meanwhile, women who gained less weight than outlined in the recommendations had offspring with increased risks of hypertension and insulin resistance, compared with those who gained weight within the recommended range (7.9 × 10^−3^ < *p* < 0.0477). Quadratic relationships for diastolic blood pressure, AUC for insulin, pancreatic beta cell function and insulin sensitivity index were confirmed in the analysis of standardised GWG (1.4 × 10^−3^ < *p*_quadratic_ < 0.0282). Further adjustment for current BMI noticeably attenuated the observed associations.

**Conclusions/interpretation:**

Both excessive and inadequate GWG have independent and significant impacts on childhood adiposity, hypertension and insulin resistance. Our findings support the notion that adverse intrauterine exposures are associated with persistent cardiometabolic risk in the offspring.

**Electronic supplementary material:**

The online version of this article (10.1007/s00125-018-4724-x) contains peer-reviewed but unedited supplementary material, which is available to authorised users.

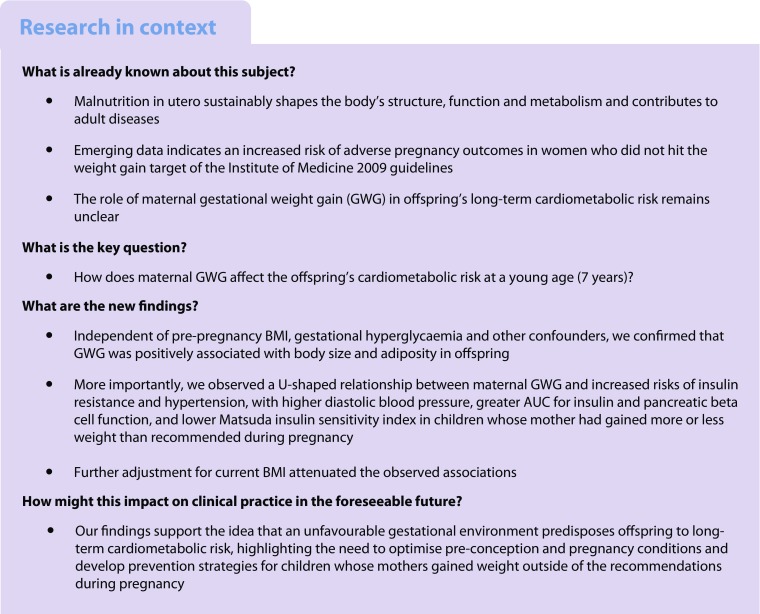



## Introduction

Type 2 diabetes has been recognised as a familial disease transmitted across generations through genetic, epigenetic and environmental circumstances including in utero exposures. To illustrate the role of early-life exposures in programming of disease, previous studies have reported evidence of an excess maternal inheritance of type 2 diabetes and cardiometabolic risk in offspring compared with the pattern of paternal inheritance [1, 2].

Barker’s hypothesis, focusing on the association of prenatal undernutrition with the risk of cardiovascular and metabolic disease in adult life, has been corroborated by the study of individuals exposed to the Dutch famine [3]. More recently, the Developmental Origins of Health and Disease (DOHaD) hypothesis extended this concept to maternal overnutrition [4]. A series of elegant studies into the Pima Indian population strongly supported this hypothesis, demonstrating the adverse effects of intrauterine exposure to hyperglycaemia on children’s adiposity and glucose metabolism [5]. One possible mechanism involved in these hypotheses is that the fetus’s adaptations to malnutrition in utero to maximise its chances for short-term survival may sustainably perturb the regulation of energy-balance and energy-sensing pathways via epigenetic modification, leading to the subsequent development of obesity and related metabolic disorders in adulthood, which may then be transmitted into the next generation [6].

With the recent upward shift of maternal weight gain during pregnancy, increasing evidence suggests a separate role for maternal gestational weight gain (GWG) on offspring cardiometabolic risk (e.g. inadequate and excessive GWG are considered to reflect undernutrition and overnutrition, respectively). The majority of studies addressing GWG in metabolic disorders have found a positive association between maternal GWG and adiposity in offspring [7, 8], although some have reported a U-shaped relationship [9, 10]. A few studies have also investigated the effect of GWG on other cardiometabolic risk factors, including fasting plasma glucose (FPG) and insulin (FPI) levels, HOMA-IR, blood pressure and lipid profile in childhood [11–13], adolescence [14] and adulthood [15–17].

To help clinicians monitor an appropriate GWG, the Institute of Medicine (IOM) reviewed the impact of weight gain during pregnancy on both maternal and fetal outcomes, and in 2009 established new guidelines for optimal GWG according to pre-pregnant BMI [18]. The IOM has, however, recognised that more research is required to study the role of maternal GWG in long-term health in the offspring.

By using follow-up data on mother–child pairs from the multicentre Hyperglycemia and Adverse Pregnancy Outcome (HAPO) study, procured at the Hong Kong study centre [19], the current study aimed to examine the relationships between maternal GWG and cardiometabolic risk factors in offspring, measured at 7 years of age, adjusting for a number of potential covariates. Birthweight is often used as an indicator of fetal conditions (e.g. growth and nutrition) in utero. It has been suggested that the relationship between birthweight and type 2 diabetes risk is U-shaped, with a higher prevalence of type 2 diabetes observed in individuals with either low or high birthweight [20]. We therefore also aimed to explore this U-shaped association.

## Methods

### Participants

**Fig. 1:**
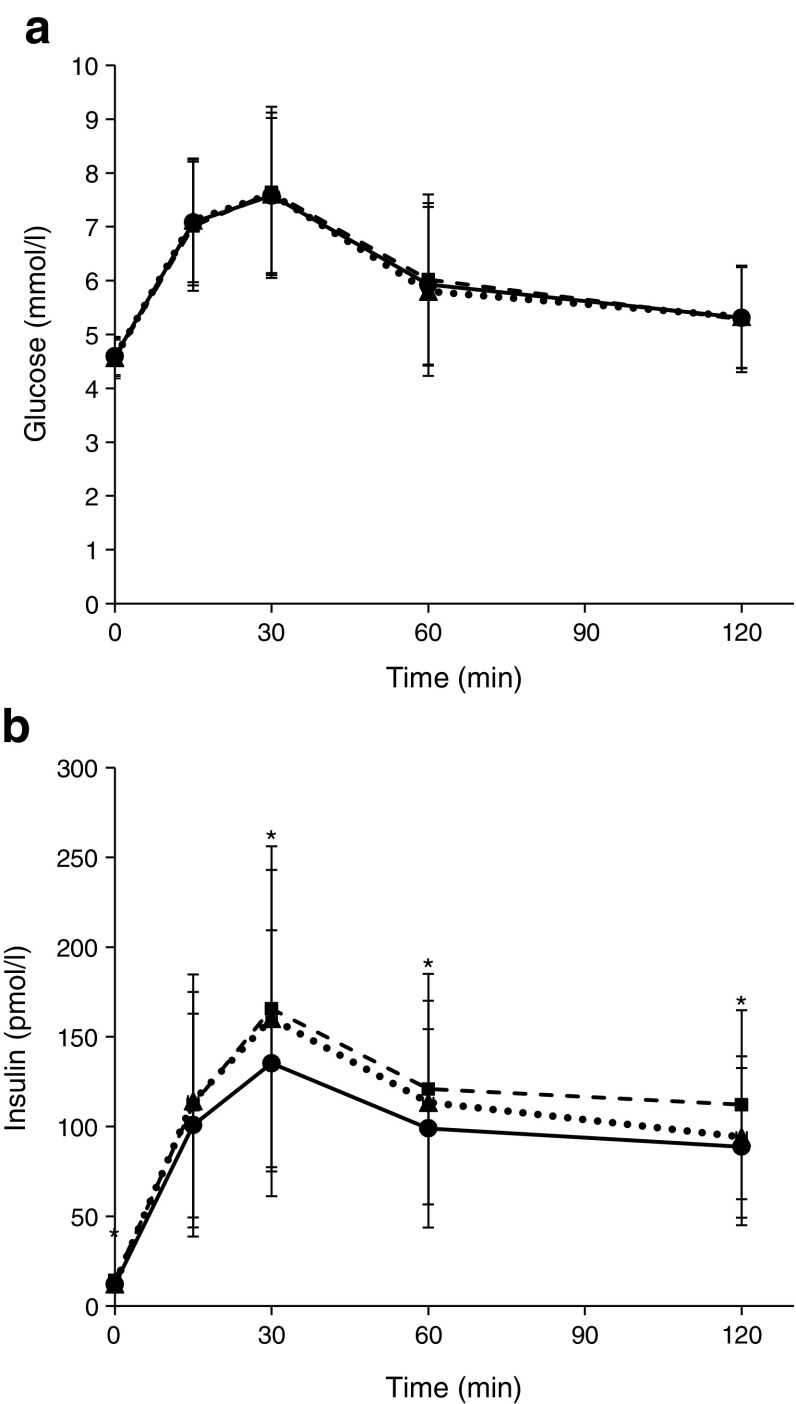
(**a**) Plasma glucose and (**b**) insulin concentrations at 0, 15, 30, 60 and 120 min during the OGTTs, stratified by IOM recommendations of GWG categories. Data are presented as mean ± SD and median ± absolute median deviation for glucose and insulin concentration, respectively, according to the time intervals. Black triangles with dotted line, group below recommendations; black circles with solid line, group within recommendations; black squares with dashed line, group exceeding recommendations. *p < 0.05 for the association across the IOM categories at each time point, with adjustments for children’s sex and age (ANOVA)

The study methods have been described previously [19]. At the Hong Kong study centre, the HAPO study recruited 1667 pregnant women with a singleton pregnancy; all mothers underwent a 75 g OGTT at 24–32 weeks’ gestation. Women of non-Chinese ancestry and women with glucose measurements beyond the setting of the HAPO study were excluded. Eligible participants were invited to attend a follow-up assessment between 2009 and 2013, when offspring were 7 years of age. The follow-up study of the children included a questionnaire, clinical examination and biochemical assessments. A total of 905 (58.2%) mother–child pairs with term pregnancies (≥37 weeks) and complete data on GWG were included for this analysis. The derivation of the eligible and analysis cohorts is shown in electronic supplementary material (ESM) Fig. 1.

This study was approved by the Clinical Research Ethics Committee of the Chinese University of Hong Kong. Research staff explained the study objective and procedures to both the mother and the child. The children’s parents or legal guardians, as appropriate, provided written informed consent.

### Exposure variables

Maternal weight at delivery was abstracted by research staff from the medical records. Pre-pregnancy BMI was calculated as self-reported pre-pregnancy weight (kg) divided by the square of the measured height (m^2^). We defined total GWG as the difference between the mother’s weight at delivery and her pre-pregnancy weight. Women were classified as gaining weight ‘below’, ‘within’, or ‘exceeding’ recommendations, according to their pre-pregnancy BMI category, on the basis of the 2009 IOM guidelines for healthy pregnancy weight gain [18].

Within each BMI category, the standardised GWG was computed as $\frac{{\mathrm{GWG}}_{ij}\hbox{---} {\mathrm{mean}}_i}{{\mathrm{SD}}_i}$ where: GWG_*ij*_ was the individual’s GWG (_*i*_ refers to the BMI categories and _*j*_ refers to each individual); mean_*i*_ was the mid-point of the range of GWG recommended by IOM in the *i*th pre-pregnancy BMI category; SD_*i*_ was (upper_*i*_ − lower_*i*_)/2; and upper_*i*_ and lower_*i*_ was the upper and lower ranges of GWG recommended by IOM in the *i*th pre-pregnancy BMI category, respectively. For example, given that the recommended range of GWG in the ‘underweight’ group is from 12.5 to 18 kg, mean_*i*_ = (18 + 12.5)/2 = 15.25 kg, and SD_*i*_ = (18–12.5)/2 = 2.75 kg.

### Outcome variables

Anthropometric indices including body weight, body height, waist and hip circumference, and biceps, triceps, subscapular and iliac skinfold thicknesses were measured. BMI was calculated as weight/height^2^. The sum of skinfold thickness was calculated as the sum of the biceps, triceps, subscapular and iliac skinfold thicknesses. Systolic and diastolic blood pressure (SBP and DBP) were measured in triplicate, and the mean readings were used. SBP and DBP for age-, sex-, and height-specific percentiles were computed based on data released from the National High Blood Pressure Education Program Working Group on High Blood Pressure in Children and Adolescents [21].

Participants were examined in the morning after an overnight fast. All individuals underwent an OGTT with a glucose load of 1.75 g/kg body weight or a full 75 g glucose load if they weighed ≥42.8 kg (i.e. 1.75 g/kg × 42.8 kg, which approximated to 75 g), with blood taken at 0, 15, 30, 60 and 120 min to measure plasma glucose and insulin levels. Plasma glucose was measured by the hexokinase method, using an automated analyser (Hitachi 911; Boehringer Mannheim, Mannheim, Germany). Plasma insulin was analysed using an immunoassay analyser (Immulite 1000 Immunoassay System; Siemens, Munich, Germany). Fasting blood samples were also collected to measure lipid profile. AUCs for glucose (AUC_glu_) and insulin (AUC_ins_) during the OGTT at 0–120 min were calculated using the trapezoid rule.

Insulin sensitivity was determined using the HOMA-IR, calculated as (FPI × FPG) ÷ 22.5 [22], and the insulin sensitivity index (ISI), estimated as 10,000 ÷ square root of [FPI × FPG × (mean glucose during OGTT) × (mean insulin during OGTT)] [23]. Beta cell function was assessed using HOMA-β, calculated as FPI × 20 ÷ (FPG − 3.5) [22]. The insulinogenic index (IGI) was calculated as (insulin during OGTT at 30 min − 0 min) ÷ (glucose during OGTT at 30 min − 0 min) [24], and pancreatic beta cell function was calculated as AUC_ins_ ÷ AUC_glu_ [25].

### Statistical analysis

All analyses were performed using either SPSS for Windows v22 (SPSS, IBM, Chicago, IL, USA) or R 2.15.1 (www.r-project.org/). *p* < 0.05 was considered statistically significant. Data are presented as percentage (*n*), mean ± SD, or median (Q1–Q3). Comparison between IOM categories of GWG was performed by *χ*^2^ test, one-way ANOVA or Kruskal-Wallis test, as appropriate.

Each normally distributed trait was winsorised by replacing any data value above the mean plus 4 SD of the sample data by the mean plus 4 SD, and any value below the mean minus 4 SD by the mean minus 4 SD. A total of 0.5% of the data were replaced. All traits were then adjusted for covariates using linear regression as follows: model 1—child’s sex, age and/or height as appropriate; model 2—model 1 + family history (maternal pre-pregnant BMI and/or maternal current hypertensive status and/or maternal and paternal current diabetes status, as appropriate); model 3—model 2 + environmental factors during prenatal (parity, maternal age, AUC_glu_ during pregnancy, mode of delivery [spontaneous, low forceps/vacuum, mid-forceps/vacuum, assisted/spontaneous breech, total breech, internal version/breech, primary Caesarean section or repeat Caesarean section] and gestational age at delivery) and postnatal periods (history of breastfeeding and childhood exercise level); model 4—model 3 + birthweight; and model 5—model 4 + childhood BMI. Individuals with missing data points for any variables included in the linear regression model were removed from the analysis. A mean of 3.9% of individuals were excluded in each regression analysis. The resulting residuals were transformed to a *z* score for normally distributed traits, and transformed to approximate normality using an inverse standard normal function for the skewed traits.

In the primary analysis, associations between IOM recommendations for GWG and transformed cardiometabolic risk factors during childhood were tested using a linear regression model. Two dummy variables were used to code for the GWG categories, ‘GWG below IOM recommendation’ and ‘GWG exceeding IOM recommendation’. Each group was compared with the reference group ‘GWG within IOM recommendation’. To investigate for non-linear associations, both linear and quadratic terms of standardised GWG were included in the regression model. The significant *p* values obtained from the regression analyses for various cardiometabolic traits are presented as a range, between the lowest and highest values among all traits. The following terminology is used for *p* values: *p*_below_, the *p* value for the comparison of GWG below the IOM recommendations with GWG within the IOM recommendations; *p*_exceeding_, the *p* value for the comparison of GWG exceeding the IOM recommendations with GWG within the IOM recommendations; *p*_linear_ and *p*_quadratic_, the *p* values of the linear and quadratic terms respectively, obtained from linear regression analyses.

## Results

### Cohort description

The characteristics of the women and newborn offspring included in this analysis are listed in ESM Table 1. Among the 905 mothers, the mean pre-pregnancy BMI was 20.9 ± 2.9 kg/m^2^, and the prevalence of women who were overweight or obese was 8.3%. The mean weight change from pre-pregnancy to delivery was 15.2 ± 4.36 kg, with 17.2% having gained weight below, 41.8% weight within and 41.0% weight exceeding the IOM recommendations. The proportions of women who gained insufficient, adequate and excessive weight differed between pre-pregnancy BMI categories (*p* = 1.4 × 10^−6^). In general, women who gained excessive weight during pregnancy were younger and had longer gestational periods, a higher primary Caesarean delivery rate and a greater BMI before pregnancy, at delivery and at 7 years after delivery. Offspring born to mothers with excessive GWG were heavier and longer, showed greater adiposity and had higher umbilical cord blood C-peptide concentrations at birth.

### IOM categories of GWG and childhood cardiometabolic risk

With adjustments for children’s sex, age and/or height, the offspring of mothers who gained excessive weight during pregnancy had a larger body size (greater height and weight), greater BMI, waist circumference and hip circumference, and higher blood pressure (higher DBP, SBP and DBP percentiles), FPI, 2 h insulin and AUC_ins_, were more insulin resistant (higher HOMA-IR and lower Matsuda ISI) and showed a greater insulin response (higher HOMA-β and IGI, and increased pancreatic beta cell function) than the offspring of mothers who achieved the weight gain target during pregnancy (4.6 × 10^−9^ < *p* < 0.0390; model 1 in Table 1; see also ESM Table 2 [model 1] and Fig. 1). Moreover, women with GWG below the IOM recommendations had children with higher DBP percentiles, AUC_ins_ and pancreatic beta cell function, and a lower Matsuda ISI, than women whose GWG was within the IOM recommendations (7.9 × 10^−3^ < *p* < 0.0477) (model 1 in Table 1; see also model 1 in ESM Table 2). Most of these associations were independent of other covariates, including maternal pre-pregnancy BMI, family histories of hypertension and diabetes in model 2, perinatal and childhood environmental factors in model 3, as well as birthweight in model 4 (Table 1). We still found significant associations between excessive GWG and body size (height and weight), adiposity traits (BMI, waist circumference and hip circumference), blood pressure (DBP and DBP percentiles), insulin levels (FPI, 2 h insulin and AUC_ins_), insulin sensitivity (Matsuda ISI) and beta cell function (HOMA-β, IGI and pancreatic beta cell function) (*p*_exceeding_ < 0.05), although the association of GWG with HOMA-IR and SBP percentile was no longer significant in models 2–4 (*p*_exceeding_ > 0.05) (Table 1). On the other hand, the association of inadequate GWG with DBP percentile (0.0279 < *p*_below_ < 0.0407) and pancreatic beta cell function (0.0184 < *p*_below_ < 0.0538) was marginally significant, while its association with AUC_ins_ (*p*_below_ = 0.0158, 0.0356, 0.0830 and 0.1357 in models 1, 2, 3 and 4, respectively) and Matsuda ISI (*p*_below_ = 0.0477, 0.0778, 0.1184 and 0.1573 in models 1, 2, 3 and 4, respectively) were slightly mitigated in models 2–4 (Table 1). However, the observed associations for both excessive and inadequate GWG in models 1–4 were notably attenuated when conditional on childhood BMI (model 5 in Table 1).

**Table 1:** Associations between IOM categories of maternal GWG and offspring cardiometabolic risk factors at 7 years of age

Phenotype	IOM category			Model 1		Model 2		Model 3		Model 4		Model 5	
	Below (*n* = 156)	Within (*n* = 378)	Exceeding (*n* = 371)	*p*_below_	*p*_exceeding_	*p*_below_	*p*_exceeding_	*p*_below_	*p*_exceeding_	*p*_below_	*p*_exceeding_	*p*_below_	*p*_exceeding_
Body size and adiposity traits													
Body height (cm)	124 ± 4.77	124 ± 4.80	125 ± 5.18	0.4773	3.1 × 10^−5^	0.6081	3.5 × 10^−4^	0.3715	7.9 × 10^−4^	0.2349	2.4 × 10^−3^	0.1925	0.0543
Body weight (kg)	22.7 ± 4.10	22.6 ± 4.06	24.5 ± 5.11	0.8822	4.6 × 10^−9^	0.8078	1.6 × 10^−6^	0.9244	1.1 × 10^−5^	0.7295	3.0 × 10^−5^	0.1963	0.0441
BMI (kg/m^2^)	14.7 ± 2.02	14.7 ± 2.01	15.6 ± 2.47	0.8635	1.3 × 10^−7^	0.5703	2.5 × 10^−5^	0.7545	1.3 × 10^−4^	0.7545	1.3 × 10^−4^	–	–
Waist circumference (cm)	53.5 ± 5.60	53.4 ± 5.47	55.4 ± 6.67	0.9405	6.0 × 10^−6^	0.7530	5.9 × 10^−4^	0.9629	2.5 × 10^−3^	0.9884	2.9 × 10^−3^	0.7543	0.4805
Hip circumference (cm)	64.2 ± 5.56	63.6 ± 5.68	66.0 ± 6.43	0.3667	4.8 × 10^−8^	0.5903	1.0 × 10^−5^	0.4230	5.2 × 10^−5^	0.3340	9.8 × 10^−5^	0.0424	0.1447
Sum of skinfold thicknesses (mm)	29.7 (23.9–39.5)	29.4 (24.7–37.7)	32.3 (25.8–44.3)	0.9372	0.0706	0.8552	0.4894	0.7172	0.6792	0.5708	0.3833	0.9580	0.1732
Blood pressure													
SBP (mmHg)	102 ± 8.76	101 ± 8.98	103 ± 8.75	0.5990	0.0729	0.7320	0.1766	0.7320	0.2728	0.9693	0.1619	0.8168	0.7175
DBP (mmHg)	62.1 ± 7.58	60.6 ± 7.86	62.9 ± 8.10	0.0513	1.4 × 10^−3^	0.0751	4.0 × 10^−3^	0.0719	8.8 × 10^−3^	0.1050	5.9 × 10^−3^	0.0446	0.0636
SBP percentile	60.6 ± 24.2	58.4 ± 24.3	63.3 ± 23.1	0.3549	0.0185	0.4531	0.0554	0.4747	0.0922	0.7029	0.0523	0.5080	0.3122
DBP percentile	62.1 ± 21.1	57.2 ± 22.1	63.0 ± 21.5	0.0186	9.7 × 10^−4^	0.0279	2.9 × 10^−3^	0.0283	5.3 × 10^−3^	0.0407	3.8 × 10^−3^	0.0157	0.0382
Glucose and insulin levels													
FPG (mmol/l)	4.57 ± 0.39	4.59 ± 0.34	4.56 ± 0.34	0.4382	0.1687	0.4135	0.1589	0.3452	0.1409	0.2862	0.1800	0.2859	0.1397
2 h glucose (mmol/l)	5.33 ± 0.95	5.31 ± 0.94	5.28 ± 0.98	0.8863	0.5970	0.9873	0.2885	0.9153	0.2314	0.8288	0.3372	0.8258	0.1555
AUC_glu_ at 0–120 min	733 ± 120	738 ± 116	740 ± 122	0.7119	0.7520	0.6424	0.9912	0.5265	0.9278	0.4221	0.8964	0.4221	0.9766
FPI (pmol/l)	12.2 (12.0–27.5)	12.0 (12.0–23.7)	14.3 (12.0–29.5)	0.2015	0.0146	0.1357	0.0105	0.2777	0.0239	0.5160	4.7 × 10^−3^	0.8403	0.2144
2 h insulin (pmol/l)	94.2 (55.7–184)	88.8 (53.8–153)	112 (66.8–182)	0.0644	1.2 × 10^−3^	0.1359	0.0184	0.2499	0.0325	0.2638	0.0249	0.1729	0.6874
AUC_ins_ at 0–120 min	13,442 (10,274–21,405)	13,122 (8070–20,669)	15,152 (9506–24,164)	0.0158	5.9 × 10^−4^	0.0356	9.8 × 10^−3^	0.0830	0.0140	0.1357	5.7 × 10^−3^	0.1712	0.3909
Beta cell function and insulin sensitivity													
HOMA-β	56.2 (40.0–92.2)	50.0 (36.4–80.0)	57.1 (40.0–98.0)	0.0689	4.2 × 10^−4^	0.0448	3.7 × 10^−4^	0.0431	2.9 × 10^−3^	0.0548	1.8 × 10^−3^	0.1010	0.1636
IGI at 30 min	58.6 (26.8–102)	47.3 (23.2–91.6)	61.0 (27.7–116)	0.1392	3.3 × 10^−3^	0.1735	0.0112	0.3117	0.0193	0.4201	0.0160	0.3748	0.2534
Pancreatic beta cell function	22.6 (16.1–36.7)	21.1 (13.1–31.8)	24.1 (15.5–38.1)	7.9 × 10^−3^	6.6 × 10^−4^	0.0184	0.0102	0.0412	0.0146	0.0538	7.5 × 10^−3^	0.0778	0.2627
HOMA-IR	0.45 (0.40–0.90)	0.43 (0.40–0.86)	0.48 (0.40–1.02)	0.2819	0.0390	0.3310	0.2309	0.3677	0.1998	0.5374	0.0741	0.7327	0.8201
Matsuda ISI	15.1 (9.78–20.6)	15.5 (10.5–22.0)	14.1 (8.56–19.1)	0.0477	6.0 × 10^−4^	0.0778	3.2 × 10^−3^	0.1184	9.8 × 10^−3^	0.1573	5.0 × 10^−3^	0.1173	0.1797

Data are expressed as percentage (*n*), mean ± SD, or median (Q1–Q3)

*p*_below_ refers to the *p* value for the comparison of GWG below the IOM recommendations with GWG within the IOM recommendations

*p*_exceeding_ refers to the *p* value for the comparison of GWG exceeding the IOM recommendations with GWG within the IOM recommendations

Model 1: adjusted for sex and age at childhood; further adjusted for childhood height for sum of skinfold thickness and blood pressure; model 2: model 1 + maternal pre-pregnant BMI; further adjusted for current maternal hypertensive status for blood pressure; or further adjusted for current maternal and paternal diabetes status for glucose and insulin levels, as well as indices for beta cell function and insulin sensitivity; model 3: model 2 + parity, maternal age, maternal AUC_glu_ during pregnancy, mode of delivery, gestational age at delivery, history of breastfeeding and childhood exercise level; model 4: model 3 + birthweight; model 5: model 4 + childhood BMI

### Standardised GWG and childhood cardiometabolic risk

To explore the non-linear/quadratic relationship between maternal GWG and childhood cardiometabolic traits, analyses were repeated using the standardised GWG. In model 1 with adjustments for children’s sex, age and/or height, there was a U-shaped relationship between standardised GWG and adverse cardiometabolic risk profile in the offspring, with higher weight, hip circumference, blood pressure (DBP and DBP percentile) and AUC_ins_, increased risks of insulin resistance (Matsuda ISI) and an enhanced insulin response (pancreatic beta cell function) in the children of women with either more or less weight gain than recommended during pregnancy (1.4 × 10^−3^ < *p*_quadratic_ < 0.0282) (model 1 in Table 2; see also model 1 in ESM Table 3 and ESM Fig. 2). Further adjustments for maternal pre-pregnancy BMI, family histories of hypertension and diabetes, perinatal and childhood environmental factors, and children’s birthweight in models 2–4 attenuated, but did not eliminate, the quadratic associations for DBP (*p*_quadratic_ = 0.0109, 0.0422, 0.0720 and 0.0808 in models 1, 2, 3 and 4, respectively), DBP percentile (*p*_quadratic_ = 6.4 × 10^−3^, 0.0254, 0.0417 and 0.0477 in models 1, 2, 3 and 4, respectively), AUC_ins_ (*p*_quadratic_ = 1.4 × 10^−3^, 0.0173, 0.0312 and 0.0381 in models 1, 2, 3 and 4, respectively), pancreatic beta cell function (*p*_quadratic_ = 3.7 × 10^−3^, 0.0302, 0.0455 and 0.0469 in models 1, 2, 3 and 4, respectively) and Matsuda ISI (*p*_quadratic_ = 4.9 × 10^−3^, 0.0401, 0.0462 and 0.0454 in models 1, 2, 3 and 4, respectively). In addition, we observed a positive linear relationship for weight (7.5 × 10^−4^ < *p*_linear_ < 0.0224 and *p*_quadratic_ > 0.05) and hip circumference (5.8 × 10^−3^ < *p*_linear_ < 0.0573 and *p*_quadratic_ > 0.05) in models 2–4 (Table 2). Only the association between standardised GWG and DBP percentile remained significant after adjusting for childhood BMI (model 5 in Table 2).

**Table 2:** Quadratic relationships between standardised maternal GWG and offspring cardiometabolic risk factors at 7 years of age

Phenotype	Standardised GWG category						Model 1		Model 2		Model 3		Model 4		Model 5	
	*z* < −3 (*n* = 15)	−3 ≤ *z* < −1 (*n* = 141)	−1 ≤ *z* < 1 (*n* = 378)	1 ≤ *z* < 3 (*n* = 260)	3 ≤ *z* < 5 (*n* = 96)	*z* ≥ 5 (*n* = 15)	*p*_linear_	*p*_quadratic_	*p*_linear_	*p*_quadratic_	*p*_linear_	*p*_quadratic_	*p*_linear_	*p*_quadratic_	*p*_linear_	*p*_quadratic_
Body size and adiposity traits																
Body height (cm)	125 ± 5.38	124 ± 4.70	124 ± 4.80	125 ± 4.75	125 ± 6.21	126 ± 5.10	0.0326	0.1301	0.0301	0.4113	0.1321	0.2836	0.3077	0.2594	0.8276	0.3230
Body weight (kg)	24.3 ± 6.16	22.5 ± 3.81	22.6 ± 4.06	24.3 ± 4.79	24.7 ± 5.71	26.5 ± 6.32	1.1 × 10^−3^	0.0183	7.5 × 10^−4^	0.3005	7.6 × 10^−3^	0.2728	0.0224	0.2528	0.9682	0.1042
BMI (kg/m^2^)	15.4 ± 2.89	14.6 ± 1.91	14.7 ± 2.01	15.5 ± 2.36	15.7 ± 2.62	16.5 ± 3.21	1.5 × 10^−3^	0.0544	1.0 × 10^−3^	0.5493	6.5 × 10^−3^	0.5771	6.5 × 10^−3^	0.5771	–	–
Waist circumference (cm)	55.6 ± 8.33	53.2 ± 5.22	53.4 ± 5.47	55.1 ± 6.31	55.6 ± 7.45	57.9 ± 7.45	9.3 × 10^−3^	0.0614	7.0 × 10^−3^	0.5838	0.0359	0.6491	0.0459	0.6309	0.6432	0.9267
Hip circumference (cm)	65.9 ± 7.76	64.0 ± 5.28	63.6 ± 5.68	65.7 ± 6.11	66.5 ± 7.11	68.1 ± 7.07	7.7 × 10^−3^	0.0282	5.8 × 10^−3^	0.3630	0.0308	0.3819	0.0573	0.3677	0.7290	0.4461
Sum of skinfold thicknesses (mm)	38.6 (24.5–46.9)	29.6 (23.9–38.8)	29.4 (24.7–37.7)	31.4 (25.7–44.5)	32.9 (26.6–43.4)	35.3 (29.9–52.6)	0.4782	0.2142	0.5463	0.9973	0.5283	0.6574	0.2726	0.6827	0.3018	0.7264
Blood pressure																
SBP (mmHg)	104 ± 10.2	101 ± 8.61	101 ± 8.98	103 ± 8.52	104 ± 9.33	105 ± 8.62	0.6948	0.2949	0.6174	0.6654	0.7601	0.7386	0.4028	0.8100	0.9667	0.8514
DBP (mmHg)	63.8 ± 8.70	62.0 ± 7.46	60.6 ± 7.86	62.5 ± 7.53	63.8 ± 9.56	65.0 ± 7.22	0.9339	0.0109	0.9518	0.0422	0.9066	0.0720	0.9026	0.0808	0.4340	0.0703
SBP percentile	64.8 ± 24.8	60.2 ± 24.1	58.4 ± 24.3	62.2 ± 23.0	65.7 ± 23.4	66.2 ± 23.2	0.6940	0.1840	0.6243	0.4674	0.7412	0.5230	0.4316	0.5848	0.9713	0.6047
DBP percentile	64.4 ± 20.8	61.9 ± 21.2	57.2 ± 22.1	62.1 ± 20.7	64.4 ± 24.0	68.1 ± 17.2	0.7079	6.4 × 10^−3^	0.7967	0.0254	0.7077	0.0417	0.8553	0.0477	0.3065	0.0410
Glucose and insulin levels																
FPG (mmol/l)	4.62 ± 0.44	4.57 ± 0.39	4.59 ± 0.34	4.54 ± 0.32	4.62 ± 0.38	4.55 ± 0.39	0.5374	0.8454	0.5916	0.9284	0.6592	0.9321	0.8100	0.9383	0.7419	0.9205
2 h glucose (mmol/l)	5.61 ± 0.61	5.30 ± 0.98	5.31 ± 0.94	5.24 ± 1.02	5.42 ± 0.88	5.01 ± 0.80	0.1962	0.6321	0.1898	0.9123	0.2322	0.6998	0.3470	0.6814	0.2130	0.6242
AUC_glu_ at 0–120 min	798 ± 98.1	726 ± 121	738 ± 116	731 ± 127	765 ± 107	747 ± 123	0.5647	0.1035	0.5461	0.1811	0.6320	0.2677	0.8482	0.2770	0.7961	0.2841
FPI (pmol/l)	12.0 (12.0–34.7)	13.7 (12.0–27.5)	12.0 (12.0–23.7)	14.4 (12.0–29.3)	14.1 (12.0–31.3)	17.0 (12.0–23.8)	0.7739	0.5006	0.8393	0.4346	0.7520	0.4338	0.2948	0.4097	0.6625	0.8614
2 h insulin (pmol/l)	93.0 (61.2–250)	94.2 (54.8–179)	88.8 (53.8–153)	108 (66.6–176)	128 (72.0–203)	121 (38.2–213)	0.5257	0.1978	0.4996	0.7464	0.4552	0.9627	0.3909	0.9832	0.4430	0.8031
AUC_ins_ at 0–120 min	15,352 (10,161–26,213)	13,410 (10,098–20,807)	13,122 (8070–20,669)	13,811 (9155–23,450)	17,588 (11,960–25,812)	21,308 (9294–24,003)	0.7073	1.4 × 10^−3^	0.6796	0.0173	0.7919	0.0312	0.8802	0.0381	0.3578	0.1260
Beta cell function and insulin sensitivity																
HOMA-β	50.0 (33.3–100)	57.1 (40.0–91.3)	50.0 (36.4–80.0)	57.1 (40.0–99.1)	50.8 (36.4–89.6)	56.6 (40.0–100)	0.4366	0.3353	0.4806	0.3867	0.9570	0.2747	0.8094	0.2636	0.4788	0.5177
IGI at 30 min	59.1 (21.9–88.9)	58.0 (27.6–105)	47.3 (23.2–91.6)	64.9 (26.9–117)	54.8 (27.9–94.3)	65.4 (34.9–107)	0.3385	0.6721	0.3555	0.9705	0.2914	0.8273	0.2166	0.8171	0.7014	0.5150
Pancreatic beta cell function	24.0 (13.9–42.1)	22.6 (16.3–35.9)	21.1 (13.1–31.8)	22.7 (15.2–38.1)	26.9 (17.4–38.1)	34.0 (14.7–47.8)	0.7008	3.7 × 10^−3^	0.6398	0.0302	0.7244	0.0455	0.9161	0.0469	0.3359	0.1016
HOMA-IR	0.42 (0.38–1.13)	0.45 (0.40–0.89)	0.43 (0.40–0.86)	0.48 (0.40–1.01)	0.47 (0.41–1.20)	0.58 (0.38–0.86)	0.9276	0.4631	0.9827	0.9872	0.9702	0.9773	0.6029	0.9403	0.8200	0.6900
Matsuda ISI	13.5 (8.31–18.9)	15.1 (9.83–20.7)	15.5 (10.5–22.0)	14.7 (8.70–20.1)	13.1 (8.14–16.9)	13.8 (8.68–18.4)	0.9534	4.9 × 10^−3^	0.9160	0.0401	0.9276	0.0462	0.8531	0.0454	0.3880	0.0638

Data are expressed as percentage (*n*), mean ± SD, or median (Q1–Q3)

*p*_linear_ and *p*_quadratic_ refer to *p* values obtained from the linear regression analysis for linear and quadratic terms, respectively

Model 1: adjusted for sex and age at childhood; further adjusted for childhood height for sum of skinfold thickness and blood pressure; model 2: model 1 + maternal pre-pregnant BMI; further adjusted for current maternal hypertensive status for blood pressure; or further adjusted for current maternal and paternal diabetes status for glucose and insulin levels, as well as indices for beta cell function and insulin sensitivity; model 3: model 2 + parity, maternal age, maternal AUC_glu_ during pregnancy, mode of delivery, gestational age at delivery, history of breastfeeding and childhood exercise level; model 4: model 3 + birthweight; model 5: model 4 + childhood BMI

## Discussion

Accumulating evidence suggests that adiposity and related cardiometabolic risk factors in the offspring are influenced by maternal weight gain during pregnancy. Using the follow-up data of 905 mother–child pairs from the HAPO study, we extended observations from earlier studies by (1) examining both linear and non-linear associations between maternal GWG and cardiometabolic risk factors in offspring aged 7 years, and (2) including data from OGTTs. To achieve a better understanding of the causal pathways, we have also systematically considered the potential confounding effects of the clinical risk factors in these relationships.

Consistent with the majority of previous studies conducted in infancy [8], early childhood [7, 11, 13] and adulthood [15–17], we confirm that maternal GWG was positively associated with body size and adiposity in offspring aged 7 years. These associations were also found to be independent of maternal pre-pregnancy BMI, shared familial genetics and environment, and environmental factors during the prenatal, perinatal and postnatal periods. Two recent studies investigated the association between pregnancy weight gain and childhood body composition assessed by dual x-ray absorptiometry (DXA) but the available evidence is inconclusive [9, 26]. Skinfold thickness, which is widely measured in children, has been suggested as a simple means of estimating body composition. In Project Viva, which included 1044 mother–child pairs, Oken et al reported an increase in the sum of skinfold thickness in the offspring at 3 years of age, along with an increase in maternal GWG [13]. However, we did not observe such association in the present study (*p*_exceeding_ = 0.0706) (model 1 in Table 1).

Several studies revealed that in utero exposure to excessive GWG predicts obesity-related cardiometabolic risk factors in both childhood [11, 13] and adulthood [15–17]. However, the existing body of literature is small and inconsistent. There are few data exploring the links between maternal GWG and insulin sensitivity and beta cell function in the offspring. In this context, we have obtained novel insights through examining the associations for insulin action and response indices calculated using OGTT data at the follow-up visit. One of the most important findings from this study was that, independently of maternal pre-pregnancy obesity and glucose level during pregnancy, maternal GWG had a U-shaped relationship with increased odds of childhood insulin resistance and hypertension, with higher DBP levels, greater AUC_ins_ and pancreatic beta cell function, and a lower Matsuda ISI in the children whose mothers gained more or less weight than recommended during pregnancy. The observed association for increased pancreatic beta cell function and reduced insulin sensitivity may reflect an initial compensation by beta cells for the obesity-induced insulin resistance, by increasing insulin secretion [27]. Similar to previous reports [11, 15–17], adjustment for childhood BMI appreciably alters these relationships.

In support of our findings, several research groups have illustrated a positive association between weight gain during pregnancy and both SBP and DBP in children [10, 11] and adults [16, 17], whereas others did not make similar observations [14, 15]. Two studies further examined the impact of maternal GWG on insulin resistance in the offspring assessed by HOMA-IR and found no evidence of association [12, 17], although Hrolfsdottir et al observed a significant association in male offspring [17]. Interestingly, none of the studies reported a U-shaped relationship between GWG and obesity-related cardiometabolic risk factors [11–13, 15–17]. The discordant findings may have arisen from the method used to calculate the total weight gain during pregnancy. As it was not always possible to obtain information on maternal weight just before conception or at delivery, the duration of the specified period for assessing GWG varies among studies. Using dynamic 5-point measurements of glucose and insulin levels during the OGTTs in this study rather than using basal measurement alone should provide a more comprehensive assessment of beta cell function and insulin sensitivity. Furthermore, the different ages of offspring participants between studies may also account for variation in findings. Larger studies are warranted to confirm the U-shaped relationship.

One potential mechanism accounting for our observed associations between GWG and cardiometabolic outcomes in the offspring is the role of shared familial genes and behaviours (e.g. diet, physical activity and socioeconomic status); for example, offspring may inherit their mother’s genetic potential to gain weight. Moreover, mothers and children may share obesogenic dietary patterns (e.g. a preference for fatty food) and lifestyle habits (e.g. lower levels of physical activity), particularly in early childhood [28], which may link greater maternal GWG with unfavourable outcomes in the offspring in later life. To take into account the factor of shared genes and behaviours, we have attempted to statistically adjust for maternal pre-pregnancy BMI, family histories of hypertension and diabetes, and characteristics reflecting the early and childhood environments. Most of the associations between GWG and childhood cardiometabolic risk were slightly attenuated but remained statistically significant, suggesting that these associations were not mainly driven by shared familial characteristics in our study. These findings point towards an alternative mechanism, implicating the potential effect of intrauterine environment.

There is increasing recognition of the potential metabolic impact of maternal adiposity, suggesting that high maternal plasma concentrations of glucose, NEFA and amino acids result in a sustained modulation of appetite control, neuroendocrine functioning or energy metabolism in the developing fetus. The fetus may thus become more vulnerable to an obesogenic environment, leading to adiposity and related cardiometabolic diseases in later life. Human studies of individuals undergoing bariatric surgery highlighted the impact of intrauterine environment on health outcomes in the offspring. A study that included 49 mothers and their 111 children aged 2.5–26 years demonstrated that offspring born after maternal biliopancreatic diversion bariatric surgery had a markedly lower prevalence of macrosomia, a reduced risk of severe obesity, greater insulin sensitivity and improved lipid profiles in adolescence, compared with their siblings born prior to the surgery [29]. In addition, Shankar et al developed a model of maternal obesity in rats based on overfeeding that allowed for a comparison in genetically identical individuals of the association between exposure to maternal obesity in utero and risk of obesity in the offspring. Shankar et al’s findings confirmed that maternal obesity at conception programmes lifelong obesity in the offspring [30]. Notably, our findings suggest that the effect of maternal GWG on childhood cardiometabolic risk is not confined to the upper and lower extremes of GWG, but rather this relationship is a U-shaped continuum. Contrary to the tenets of Barker’s hypothesis, which focuses on extreme prenatal challenges as being crucial to the disease pathway, a new global landscape of disease is emerging in which the relationship between developmental environment and disease risk is a U-shaped continuum within the physiological range [31]. It also suggests that subtle variations in prenatal experience (e.g. maternal nutrition) might affect the risk of disease, and this effect can be amplified at all levels by a plentiful environment (e.g. unhealthy diet and lifestyle) in adulthood [31]. The lowest risk of disease occurs when both developmental and adult environments are optimal.

The current study has unique strengths. Our mother–child cohort was prospectively designed, with comprehensive clinical assessments of childhood cardiometabolic risk factors including glucose and insulin levels during OGTTs. The availability of detailed measures collected in early life and childhood permitted meaningful and stepwise adjustment for a large number of potential confounders. Owing to the design of the HAPO study, the mothers and their doctors were blinded to the OGTT results and hence did not receive any dietary or medical treatment for hyperglycaemia during pregnancy, which would have confounded similar analyses in other studies.

There are, however, several limitations of the study. First, pre-pregnancy weight was self-reported by the mothers, and thus the estimation of GWG may be inaccurate. We noted that there was a strong correlation between the self-reported pre-pregnancy weight and the weights measured at the pregnancy OGTT visits (*r*^2^ = 0.899), supporting the validity of the self-reported data. Second, the IOM recommendations for GWG were developed in a cohort consisting largely of healthy white women, and used the standard BMI thresholds to define the categories of GWG. As adults in Asia have different BMI classification thresholds for overweight and obesity, further studies with sufficiently large sample sizes are required to determine the optimal GWG for Asian populations. Third, 41.8% (*n* = 697) of participants were non-eligible, declined the follow-up visit or were not contactable. Compared with children who attended the follow-up visit, we found no evidence of discrepancy in the distributions of GWG between two groups (ESM Table 4). Therefore, the observed associations are unlikely to be due to selection bias. Fourth, our findings were restricted to individuals of Chinese ancestry and children aged 6–8 years, with limited generalisability. Last, we were not able to explore the associations using GWG at different stages of pregnancy because repeated measures of maternal weight were not recorded in this study.

In conclusion, we found evidence of linkage between GWG and several cardiometabolic risk factors in the offspring aged 7 years, independent of maternal BMI prior to pregnancy and glucose levels during pregnancy. These findings have important implications for prevention and treatment. Pregnancy may be a potential window of opportunity for intervention through modifiable behaviours, including maternal nutrition and physical activity. Although limiting excessive GWG may help minimise the intergenerational cycle of obesity, the benefits of lower weight gain must be balanced against other cardiometabolic risks (e.g. hypertension and insulin resistance) and risk of stunted growth in the offspring if maternal GWG is inadequate. Finally, long-term follow-up of these children is necessary to evaluate the effect of maternal GWG on cardiometabolic risk in adolescence and adulthood.

## Electronic supplementary material

ESM (PDF 1.82 mb)

## Data Availability

The data supporting the findings of this study are available on request from the corresponding authors (WHT and RCWM). The data are not publicly available as they contain information that could compromise the privacy or consent of the research participants.

## Abbreviations

AUC_glu_: AUC for glucose
AUC_ins_: AUC for insulin
DBP: Diastolic blood pressure
FPG: Fasting plasma glucose
FPI: Fasting plasma insulin
GWG: Gestational weight gain
HAPO: Hyperglycemia and Adverse Pregnancy Outcome
IOM: Institute of Medicine
ISI: Insulin sensitivity index
IGI: Insulinogenic index
SBP: Systolic blood pressure
