# Supplementary material for: The impact of maternal gestational weight gain on cardiometabolic risk factors in children
Source: Diabetologia. 2018 Sep 17;61(12):2539–48. doi: 10.1007/s00125-018-4724-x (PMC6223878; doi:10.1007/s00125-018-4724-x)
Supplement: Supplementary file 1 — (PDF 1.82 mb) [file 125_2018_4724_MOESM1_ESM.pdf]

ESM Table 1. Maternal and newborn characteristics according to IOM categories of maternal gestational weight gain.

		IOM category			
Phenotype	Total	Below	Within	Exceeding	p
N (%)	905 (100%)	156 (17.2%)	378 (41.8%)	371 (41.0%)	
**Maternal characteristics**					
During pregnancy					
Age (year)	31.3 ± 4.64	32.3 ± 4.65	31.3 ± 4.42	30.8 ± 4.73	2.0×10^{-3}
Prenatal smoking (%)	1.5% (14)	0.6% (1)	1.1% (4)	2.4% (9)	0.1906
Prenatal alcohol use (%)	0.1% (1)	0.0% (0)	0.0% (0)	0.3% (1)	0.4865
Parity (%)					
0	59.8% (541)	45.5% (71)	63.2% (239)	62.3% (231)	
1	32.8% (297)	46.2% (72)	29.9% (113)	30.2% (112)	1.9×10^{-3}
≥2	7.4% (67)	8.3% (13)	6.9% (26)	7.5% (28)	
Glucose AUC at OGTT 0 – 120 min during pregnancy	798 ± 130	790 ± 108	783 ± 133	793 ± 136	0.5437
Pre-pregnancy body mass index category (%)					
Underweight (<18.5 kg/m^2)	18.2% (165)	23.1% (36)	23.8% (90)	10.5% (39)	
Normal weight (18.5 - 24.9 kg/m^2)	73.5% (665)	71.8% (112)	70.9% (268)	76.8% (285)	1.4×10^{-6}
Overweight (25.0 - 29.9 kg/m^2)	7.3% (66)	3.8% (6)	5.0% (19)	11.1% (41)	
Obese (≥30 kg/m^2)	1.0% (9)	1.3% (2)	0.3% (1)	1.6% (6)	
Pre-pregnancy body mass index (kg/m^2)	20.9 ± 2.85	20.8 ± 2.83	20.4 ± 2.59	21.4 ± 3.00	2.1×10^{-6}
Body mass index at delivery (kg/m^2)	27.0 ± 3.10	24.6 ± 2.40	26.0 ± 2.23	29.0 ± 2.84	9.4×10^{-77}
Weight gain from pre-pregnancy to delivery (kg)	15.2 ± 4.36	9.34 ± 2.44	13.9 ± 1.87	19.0 ± 3.09	2.5×10^{-214}
Percentage change of weight from pre-pregnancy to delivery	29.7 ± 9.38	18.7 ± 5.73	28.1 ± 6.15	36.0 ± 8.34	2.4×10^{-112}
Pregnancy outcomes					
Primary cesarean delivery (%)	18.7% (169)	9.6% (15)	18.3% (69)	22.9% (85)	1.6×10^{-3}
Pre-eclampsia (%)	1.5% (14)	0.0% (0)	1.9% (7)	1.9% (7)	0.2273

Gestational age at delivery (weeks)	39.6 ± 1.13	39.3 ± 1.10	39.6 ± 1.12	39.7 ± 1.13	3.2×10^{-3}
During follow-up					
Body mass index (kg/m^2)	23.1 ± 3.51	22.1 ± 3.20	22.4 ± 3.11	24.2 ± 3.65	2.5×10^{-16}
Diabetes (%)	1.2% (11)	1.9% (3)	1.6% (6)	0.5% (2)	0.2875
Spouse diagnosed with diabetes (%)	2.5% (23)	2.6% (4)	2.7% (10)	2.4% (9)	0.9796
Hypertension (%)	5.2% (47)	7.7% (12)	4.2% (16)	5.1% (19)	0.2638
**Newborn characteristics**					
Sex (male%)	51.9% (470)	53.2% (83)	51.3% (194)	52.0% (193)	0.9237
Birthweight (g)	3220 ± 392	3065 ± 375	3181 ± 369	3325 ± 397	7.5×10^{-13}
Z-score of customized birthweight	-0.03 ± 1.07	-0.35 ± 0.96	-0.06 ± 1.02	0.14 ± 1.11	5.4×10^{-6}
Large for gestational age (%)	10.5% (95)	1.9% (3)	9.3% (35)	15.4% (57)	1.5×10^{-5}
Birth length (cm)	49.3 ± 1.75	48.9 ± 1.71	49.2 ± 1.72	49.5 ± 1.77	3.3×10^{-4}
Flank skinfold (mm)	3.20 ± 0.83	3.02 ± 0.77	3.14 ± 0.73	3.34 ± 0.91	9.2×10^{-5}
Triceps skinfold (mm)	4.42 ± 0.87	4.21 ± 0.80	4.35 ± 0.84	4.58 ± 0.91	9.9×10^{-6}
Subscapular skinfold (mm)	4.29 ± 0.93	4.02 ± 0.76	4.24 ± 0.85	4.46 ± 1.05	4.7×10^{-6}
Sum of skinfold thickness (mm)	11.9 ± 2.30	11.3 ± 2.00	11.7 ± 2.08	12.4 ± 2.54	4.9×10^{-7}
Body fat percentage	9.88 ± 3.27	8.55 ± 3.11	9.54 ± 3.27	10.7 ± 3.12	2.1×10^{-12}
Cord blood C-peptide (pmol/l)	298 (232-430)	265 (232-397)	298 (232-397)	331 (265-430)	0.0150
Neonatal clinical hypoglycaemia (%)	4.1% (37)	4.5% (7)	3.7% (14)	4.3% (16)	0.8809

Data were expressed as percentage (n), mean ± SD, or median (Q1-Q3). Sum of skin folds at birth was calculated as the sum of flank, triceps and subscapular skin fold thicknesses. To test the differences among IOM category of maternal gestational weight gain, *p*-values were obtained from χ^2 test for categorical variables and ANOVA or Kruskal-Wallis test for continuous variables. AUC, area under the curve.

ESM Table 2. Associations between IOM categories of maternal gestational weight gain and offspring's lipid profiles at 7 years of age.

	IOM category			Model 1		Model 2		Model 3		Model 4		Model 5	
Phenotype	Below	Within	Exceeding	p_{below}	$p_{exceeding}$	p_{below}	$p_{exceeding}$	p_{below}	$p_{exceeding}$	p_{below}	$p_{exceeding}$	p_{below}	$p_{exceeding}$
Lipids profile													
Total cholesterol (mmol/l)	4.47 ± 0.73	4.48 ± 0.73	4.46 ± 0.69	0.8512	0.7568	0.7774	0.5434	0.8662	0.4747	0.9606	0.3425	0.9617	0.3026
Triglycerides (mmol/l)	0.70 (0.50 - 0.90)	0.70 (0.50 - 0.80)	0.70 (0.50 - 0.90)	0.8794	0.7647	0.9717	0.9919	0.9473	0.9011	0.8861	0.8822	0.7707	0.3961
HDL-cholesterol (mmol/l)	1.65 ± 0.30	1.67 ± 0.35	1.65 ± 0.35	0.5922	0.5215	0.6001	0.5487	0.6950	0.6119	0.7200	0.5426	0.7086	0.9914
LDL-cholesterol (mmol/l)	2.48 ± 0.66	2.48 ± 0.62	2.47 ± 0.59	0.9449	0.7161	0.8632	0.4905	0.9522	0.4162	0.9551	0.3072	0.9514	0.1982

Data were expressed as percentage (n), mean ± SD, or median (Q1-Q3). P_{below} refers to the *p*-value for the comparison of GWG below IOM recommendation with GWG within IOM recommendation. $P_{exceeding}$ refers to the *p*-value for the comparison of GWG exceeding IOM recommendation with GWG within IOM recommendation. Model 1: adjusted for sex and age at childhood; Model 2: Model 1 + maternal prepregnant BMI; Model 3: Model 2 + parity, maternal age, maternal AUC_{glu} during pregnancy, mode of delivery, gestational age at delivery, history of breast feeding, and childhood exercise level; Model 4: Model 3 + birthweight; Model 5: Model 4 + childhood BMI.

ESM Table 3. The quadratic relationships between standardized maternal gestational weight gain and offspring's lipid profiles at 7 years of age.

	Standardized gestational weight gain category						Model 1		Model 2		Model 3		Model 4		Model 5	
Phenotype	z <-3	-3 ≤ z < -1	-1 ≤ z < 1	1 ≤ z < 3	3 ≤ z < 5	z ≥5	p_{linear}	$p_{quadratic}$	p_{linear}	$p_{quadratic}$	p_{linear}	$p_{quadratic}$	p_{linear}	$p_{quadratic}$	p_{linear}	$p_{quadratic}$
<u>Lipids profile</u>																
Total cholesterol (mmol/l)	4.74 ± 0.81	4.44 ± 0.71	4.48 ± 0.73	4.45 ± 0.66	4.52 ± 0.77	4.30 ± 0.65	0.5641	0.5450	0.5658	0.7808	0.5211	0.9186	0.3727	0.8936	0.3456	0.9055
Triglycerides (mmol/l)	0.60 (0.50 - 1.10)	0.70 (0.50 - 0.88)	0.70 (0.50 - 0.80)	0.70 (0.50 - 0.90)	0.70 (0.50 - 0.90)	0.70 (0.50 - 1.00)	0.8189	0.5167	0.8490	0.7433	0.8605	0.9322	0.7980	0.9467	0.3676	0.9860
HDL-cholesterol (mmol/l)	1.64 ± 0.32	1.65 ± 0.29	1.67 ± 0.35	1.66 ± 0.35	1.64 ± 0.34	1.60 ± 0.33	0.4794	0.1380	0.4786	0.1497	0.5164	0.1984	0.5929	0.2131	0.3541	0.2500
LDL-cholesterol (mmol/l)	2.79 ± 0.81	2.45 ± 0.64	2.48 ± 0.62	2.45 ± 0.56	2.52 ± 0.68	2.38 ± 0.63	0.2876	0.2204	0.2881	0.3838	0.2385	0.4731	0.1620	0.4614	0.1167	0.4901

Data were expressed as percentage (n), mean ± SD, or median (Q1-Q3). P_{linear} and $P_{quadratic}$ refer to p-values obtained from the linear regression analysis for linear and quadratic terms, respectively. Model 1: adjusted for sex and age at childhood; Model 2: Model 1 + maternal prepregnant BMI; Model 3: Model 2 + parity, maternal age, maternal AUC_{glu} during pregnancy, mode of delivery, gestational age at delivery, history of breast feeding and childhood exercise level; Model 4: Model 3 + birthweight; Model 5: Model 4 + childhood BMI.

ESM Table 4. Comparison of baseline maternal and offspring characteristics stratified according to follow-up status.

Phenotype	Included in follow-up study: No	Included in follow-up study: Yes	*p*
N (%)	697 (41.8%)	970 (58.2)	--
**Maternal characteristics**			
During pregnancy			
Age (year)	30.4 ± 5.17	31.3 ± 4.64	2.9×10^{-4}
Prenatal Smoking (%)	2.8% (19)	1.6% (16)	0.1206
Prenatal Alcohol Use (%)	0.1% (1)	0.2% (2)	0.7740
Parity (%)			
0	63.1% (435)	59.5% (577)	
1	29.5% (203)	33.3% (323)	0.2505
≥2	7.4% (51)	7.2% (70)	
Glucose AUC at OGTT 0 – 120 min during pregnancy	786 ± 174	791 ± 132	0.4043
Pre-pregnancy body mass index category (%)			
Underweight (<18.5 kg/m^2)	24.3% (165)	18.9% (182)	
Normal weight (18.5 - 24.9 kg/m^2)	67.4% (458)	72.7% (700)	0.0645
Overweight (25.0 - 29.9 kg/m^2)	7.6% (52)	7.5% (72)	
Obese (≥30 kg/m^2)	0.7% (5)	0.9% (9)	
Pre-pregnancy body mass index (kg/m^2)	20.5 ± 2.89	20.9 ± 2.85	3.7×10^{-3}
Body mass index at delivery (kg/m^2)	26.7 ± 3.34	26.9 ± 3.12	0.1187
Weight gain from pre-pregnancy to delivery (kg)†	15.6 ± 4.9	15.1 ± 4.36	0.1055
IOM categories of gestational weight gain (%)			
Below	18.5% (123)	18.5% (175)	
Within	38.9% (259)	41.4% (392)	0.5795
Exceed	42.6% (283)	40.2% (381)	
Percentage change of weight from pre-pregnancy to delivery†	30.9 ± 10.4	29.6 ± 9.37	0.1333
Pregnancy outcomes			
Primary cesarean delivery (%)	21.4% (147)	19.2% (186)	0.2726
Pre-eclampsia (%)	3.1% (21)	1.9% (18)	0.1132
Gestational age at delivery (weeks)	39.3 ± 1.5	39.4 ± 1.5	0.7860
**Newborn characteristics**			
Sex (male%)	47.2% (325)	48.2% (468)	0.6650
Birthweight (g)	3146 ± 418	3180 ± 436	0.1100
Z-score of customized birthweight	-0.11 ± 1.07	-0.03 ± 1.07	0.1605
Large for gestational age (%)	10.7% (73)	10.5% (101)	0.8852

Birth length (cm)	49.1 ± 1.96	49.2 ± 1.83	0.4805
Flank skinfold (mm)	3.18 ± 0.84	3.2 ± 0.82	0.6601
Triceps skinfold (mm)	4.37 ± 0.88	4.41 ± 0.87	0.3813
Subscapular skinfold (mm)	4.23 ± 1	4.28 ± 0.93	0.2889
Sum of skinfold thickness (mm)	11.8 ± 2.37	11.9 ± 2.29	0.3471
Body fat percentage	9.5 ± 3.23	9.76 ± 3.33	0.1183
Cord blood C-peptide (pmol/l)	298 (232-397)	298 (232-430)	0.0191
Neonatal clinical hypoglycaemia (%)	6.3% (43)	4.8% (47)	0.2136

Data were expressed as mean ± SD or median (Q1 - Q3). Sum of skin folds at birth was calculated as the sum of flank, triceps and subscapular skin fold thicknesses. To test the difference among group, *p*-values were obtained from χ^2 test for categorical variables and ANOVA or Mann Whitney U test for continuous variables. "†", weight gain from pre-pregnancy to delivery and glucose AUC at OGTT 0-120 min during pregnancy were adjusted for maternal pre-pregnancy BMI.

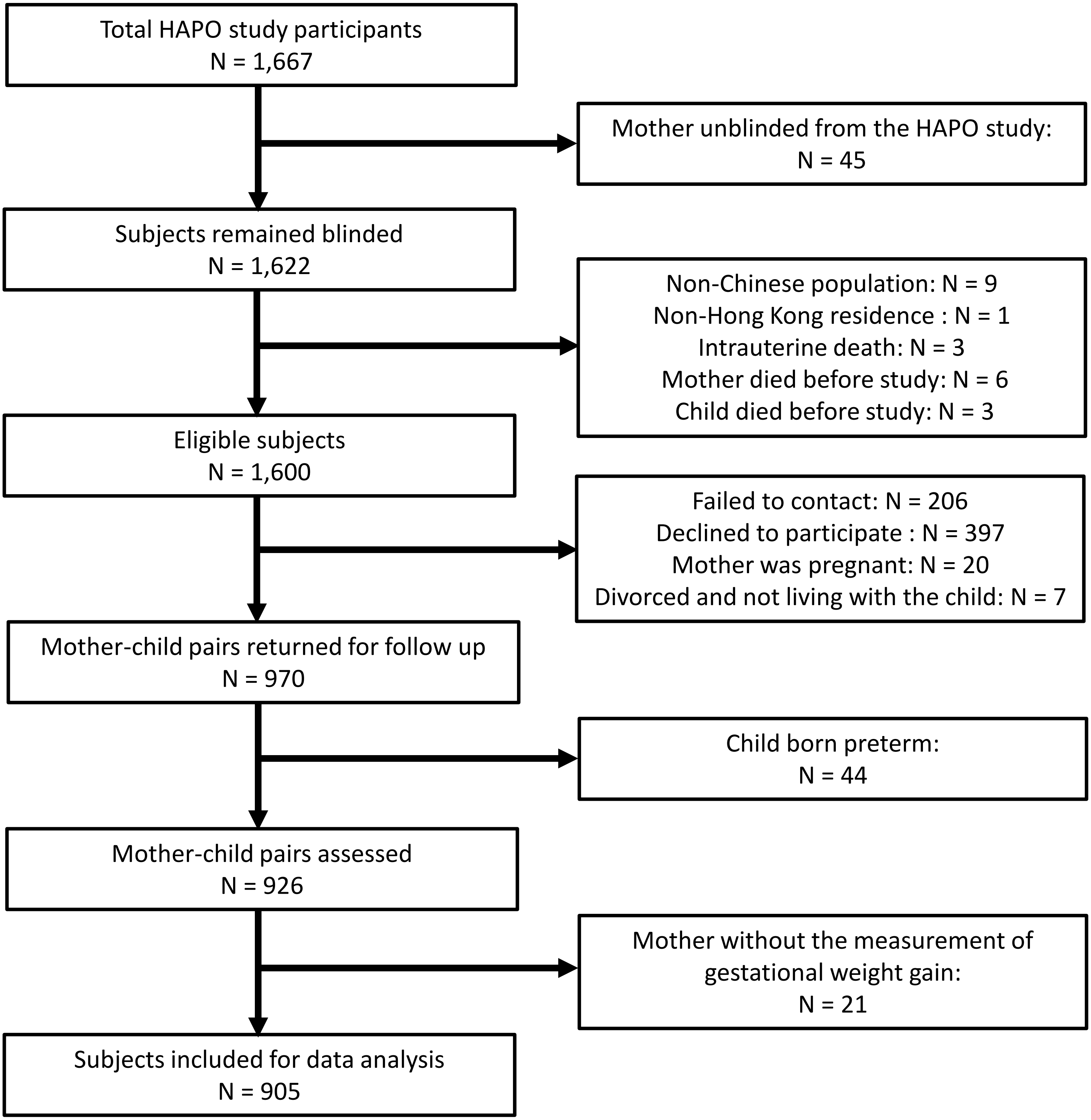

ESM Fig. 1. Number of subjects included in the analyses.

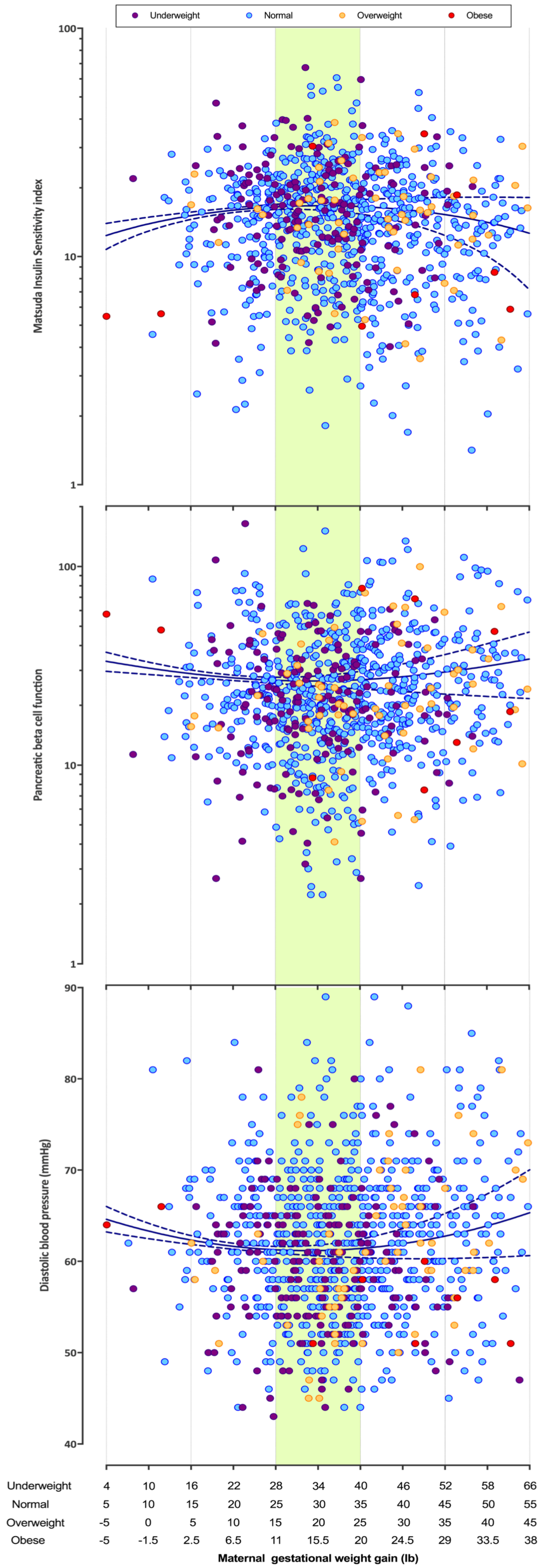

ESM Fig. 2. The non-linear relationships between maternal gestational weight gain and (a) diastolic blood pressure, (b) Matsuda insulin sensitivity index and (c) Pancreatic Beta cell function were fitted by a quadratic curve with 95% confidence interval, after adjusting for the categories of pre-pregnancy BMI.
